# SUBJECTIVE EXPERIENCES OF AGING AND PHYSICAL ACTIVITY IN THE YEARS THAT PRECEDE AND FOLLOW A FALL

**DOI:** 10.1093/geroni/igac059.379

**Published:** 2022-12-20

**Authors:** Shannon Mejía, Tai-Te Su, Faith-Christina Washington

**Affiliations:** University of Illinois at Urbana-Champaign, Champaign, Illinois, United States; The University of Illinois Urbana-Champaign, Champaign, Illinois, United States; University of Illinois at Urbana-Champaign, Champaign, Illinois, United States

## Abstract

Falls are the leading preventable cause of death and disability in older adulthood. Subjective experiences of aging could facilitate fall prevention and adaptation to post-falls life. We use data from the 2008-2018 waves of the Health and Retirement Study to follow self-perceptions of aging (SPA), health-domain control (HDC), and physical activity (PA) in 12,000+ adults (Mage=69.09; 59% women; 83% white) to examine trajectories of subjective experiences and health behaviors preceding and following a fall. In total, 57% experienced falling. Both SPA and HDC were lower among fallers. Spline growth models showed that HDC, SPA, and PA significantly decreased over time. Additionally, the rate of decline in HDC and PA increased following the fall. After falling, the protective effect of HDC amplified, while positive SPA dampened change in PA. Our study illustrates the importance of subjective experiences of aging on adaptation and recovery in the context of falling.

